# Ca^2+^ channels in retinal pigment epithelial cells regulate vascular endothelial growth factor secretion rates in health and disease

**Published:** 2007-03-27

**Authors:** Rita Rosenthal, Heinrich Heimann, Hansjürgen Agostini, Gottfried Martin, Lutz Lothar Hansen, Olaf Strauss

**Affiliations:** 1 Institut für Klinische Physiologie, Charité-Universitatsmedizin Berlin, Campus Benjamin Franklin, Berlin, Germany; 2 St. Pauls Eye Unit, Royal Liverpool Hospital, Liverpool, United Kingdom; 3 Augenklinik des Universitätsklinikums Freiburg, Albert-Ludwigs-Universität Freiburg, Freiburg, Germany; 4 Experimentelle Ophthalmologie, Klinik und Poliklinik für Augenheilkunde, Universitätsklinikum Hamburg-Eppendorf, Germany

## Abstract

**Purpose:**

Choroidal neovascularization (CNV) is the most severe complication in age-related macular degeneration. The major angiogenic factor involved is vascular endothelial growth factor (VEGF) secreted by the retinal pigment epithelium (RPE). Since RPE cells express neuroendocrine L-type Ca^2+^ channels we investigated their involvement in VEGF secretion in normal RPE cells and RPE cells from patients with CNV.

**Methods:**

Freshly isolated and cultured RPE cells were studied using the patch-clamp technique and ELISA-based secretion assays.

**Results:**

Both freshly isolated and cultured cells showed whole-cell Ba^2+^ currents with properties of L-type Ca^2+^ currents: high activation threshold, sensitivity to dihydropyridines (10 μM nifedipine) and slow inactivation. VEGF-A secretion was elevated by BayK8644 (10 μM) or basic fibroblast growth factor (bFGF, 10 ng/ml), both of which are able to activate L-type channels. Cells from CNV tissue also showed nifedipine-sensitive Ba^2+^ currents, which displayed a voltage-dependent activation at more negative potentials, faster inactivation and changed regulation by tyrosine kinase pp60^c-src^. The CNV RPE cells showed higher VEGF secretion rates which were reduced by nifedipine.

**Conclusions:**

Thus, L-type Ca^2+^ channels in normal RPE cells regulate the secretion of VEGF. RPE cells from eyes with CNV maintain a VEGF secretion regulated by nifedipine-sensitve Ca^2+^ channels which might be of importance for the development of CNV.

## Introduction

The retinal pigment epithelium (RPE) is a monolayer of pigmented cells that closely interacts with photoreceptors to maintain their structural integrity and excitability [1-4]. A changed growth factor secretion by the RPE is believed to be involved in the etiology of proliferative eye diseases,such as choroidal neovascularization (CNV) [5-8] in age-related macular degeneration (AMD), which is the most common cause for legal blindness in industrialized countries [9]. Mainly a changed growth factor secretion by the RPE seems to be of importance for initiating CNV: increased secretion of pro-angiogenic factors and decreased secretion of anti-angiogenic factors. CNV, representing the most severe complication in AMD, originates in choroidal blood vessels that grow through Bruch's membrane into the sub RPE as well as the subretinal space. In many studies, but especially in recent clinical trials, vascular endothelial growth factor (VEGF) appears to be the major angiogenic factor in this process.

Several studies using cultured or freshly isolated RPE cells from various species demonstrated the expression of L-type Ca^2+^ channels [10-15]. L-type Ca^2+^ channels represent a group of high-voltage activated Ca^2+^ channels [16,17]. Studies examining the regulation of L-type channels of the RPE suggested that these Ca^2+^ channels provide a Ca^2+^-influx pathway involved in growth factor-dependent intracellular signaling [12,15]. Activation of L-type channels in the RPE by the cytosolic subtype tyrosine kinase pp60^c-src^ shifts the voltage-dependent activation to a more negative voltage-range, closer to the resting potential of RPE cells [12,18]. This leads to a higher number of active channels and, thus, an increase in intracellular free Ca^2+^. In this way, high-voltage-activated Ca^2+^ channels can contribute to intracellular signaling in epithelial cells. A voltage-dependent activation in a rather negative voltage-range has been discussed as a property of L-type channels composed of Ca_v_1.3 α-subunits [19-21]. The expression of Ca_v_1.3 subunits has been demonstrated in rat RPE cells [12,15]. Thus, L-type channels in RPE cells can participate in intracellular signaling which could mediate changes in a yet unknown RPE cell function.

L-type channels composed of the Ca_v_1.3 α-channel-subunit belong to the neuroendocrine subtype, because these channels are known to regulate the insulin secretion by β-islet cells in the pancreas [16]. The RPE is known to secrete a variety of growth factors like insulin like growth factor-1 (IGF-1), basic fibroblast growth factor (bFGF or FGF2), VEGF or pigment epithelium-derived factor (PEDF) [7,22-30]. In order to show that L-type channels might regulate secretion rates in RPE cells we studied voltage-dependent Ca^2+^ channels and the regulation of VEGF-A secretion by human RPE cells and by RPE cells from surgically excised CNV tissues of AMD patients. We found that VEGF secretion is dependent on the activity of voltage-dependent Ca^2+^ channels, which might provide new targets to prevent CNV by interventions at the source for VEGF.

## Methods

### Human tissue

For the use of human material, tenets of the Declaration of Helsinki were followed, informed consent was obtained, and Institutional Human Experimentation Committee approval was granted for the studies. CNV membranes were obtained directly after eye surgery of patients with AMD. The mean and the range of the age of the patients are given in Table 1. Patients with severe systemic diseases were not included in the study. The human tissue was obtained about one hour after eye surgery or enucleation and immediately prepared for the experiments The detailed information is listed in Table 1.

**Table 1:** Origin of human tissue.

Patient information	RPE	CNV
Number of patients	7	18
Mean age of patients	69.0±1.6	70.7±4.0
Male	3	7
Female	4	11

Retinal pigment epithelium (RPE) was isolated from eyes without choroidal neovascularization (CNV). These eyes were obtained from multi organ donors after removal of cornea for transplantation. CNV is derived from choroidal neovascular tissue of patients with age-related macular degeneration (AMD); removal of CNV tissue was indicated by individuell clinical picture. All AMD patients suffered from wet AMD. Since only 46% of RPE cells in CNV tissue express voltage-dependent channels, a larger number of patients is included in this study.

### Patch-clamp recordings with freshly isolated cells

A portion of the CNV membranes was dissolved into single cell suspension by enzymatic digestion using protease (10%) and trypsin (0.1%). Cells from donor eyes without CNV were placed into single-cell suspension by the same method. Cell suspensions were placed onto a poly-lysine-coated glass cover slip and allowed to settle. The cover slips were placed into the perfusion chamber on the stage of an inverted microscope and constantly superfused. The bath solution contained the following (given in mM): 136.4 NaCl, 1.1 NaH_2_PO_4_, 4.2 NaHCO_3_, 0.9 MgCl_2_, 0.95 CaCl_2_, 10.0 BaCl_2_, 5.8 TEACl, 25.0 HEPES, and 11.1 glucose. Perforated-patch recordings were performed with patch-pipettes of 3-5 mega-Ω resistance, using a pipette solution that contained the following (given in mM): 100.0 CsCl, 10.0 NaCl, 2.0 MgSO_4_, 0.5 CaCl_2_, 5.5 EGTA, 10.0 HEPES, nystatin (150 mg/ml). For perforated-patch recordings from pigmented cells, we added an antibody against class III β-tubulin (Sigma, Deisenhofen, Germany) to the pipette solution. Currents were measured using an EPC-7 patch-clamp amplifier in conjunction with TIDA hardware and software (HEKA, Lamprecht, Germany). The freshly isolated cells showed a membrane capacitance of 43.3±6.9 pF (n=10), and access resistance of 12.5±2.0 mega-Ω (n=10). Access resistance was compensated to values lower than 10 mega-Ω. Seals were stable for approximately 3-7 min.

**Figure 1:**
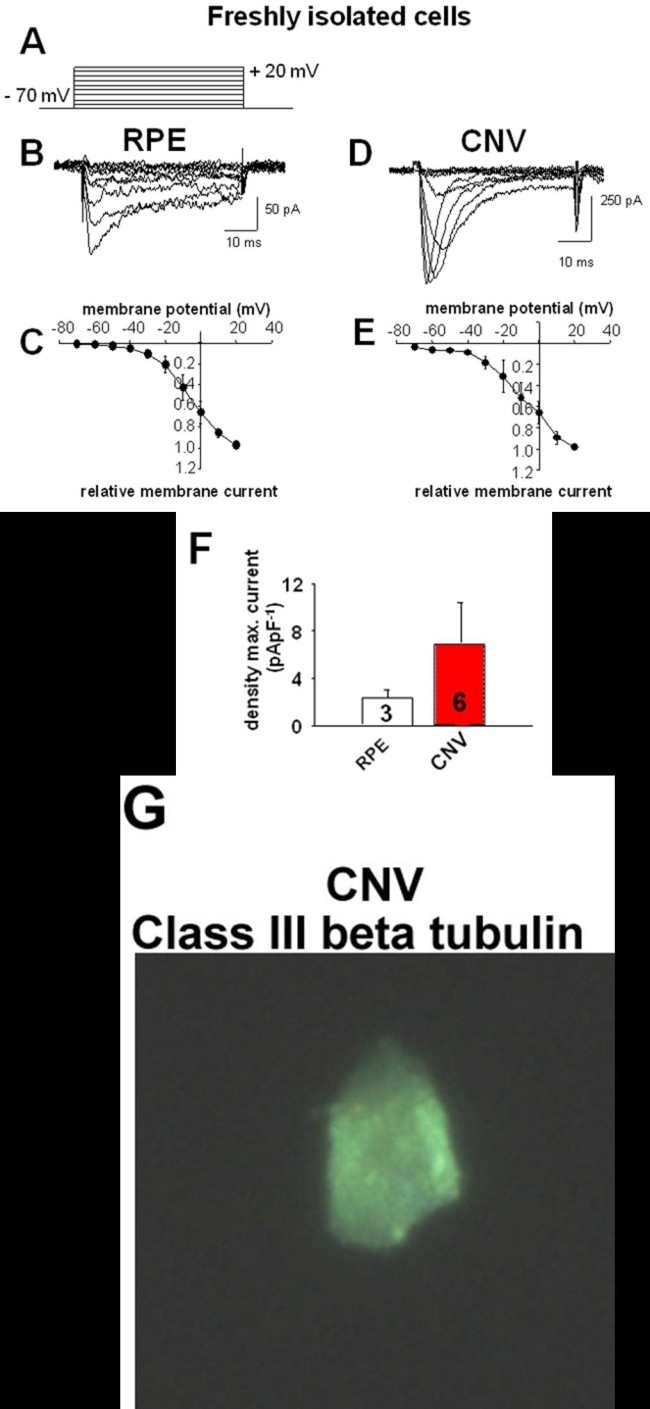
Voltage-dependent Ba^2+^ currents in freshly isolated cells. **A**: Pattern of electrical stimulation to activate voltage-dependent Ba^2+^ currents. **B**: Ba^2+^ inward currents in a human retinal pigment epithelium (RPE) cell freshly isolated from an eye without choroidal neovascularization (CNV). **C**: Current/voltage plot of currents from three freshly isolated cells from eyes without CNV **D**: Ba^2+^ inward currents in a human RPE cell freshly isolated from CNV tissue. **E**: Current/voltage plot of currents from six freshly isolated cells from CNV membranes. **F**: Comparison of maximal Ba^2+^ densities at +10 mV in freshly isolated cells. **G**: Class III β-tubulin staining of a freshly isolated cell from CNV membrane after patch-clamp recording.

For stimulation of Ba^2+^ currents through L-type channels cells were depolarized from a holding potential of -70 mV by 9 voltage-steps of 50 ms duration up to +20 mV (Figure 1A). Measurements of maximal current density and kinetic parameters occurred at +10 mV to ensure data comparison at equal biophysical driving forces.

### Patch-clamp recordings from cultured cells

CNV membrane pieces not used for in situ investigations were employed for growing RPE cell cultures, using the method of Schlunck et al. [31]. Cultures from eyes without CNV were established using the method of Aronson [32]. Recordings from cultured cells were performed under the same conditions with the same pipette and bath solutions as used for freshly isolated cells. Purity of cell cultures was confirmed by immunohistochemical staining for class III β-tubulin, vimentin, cytokeratin 18, glial fibrillary acidic protein (GFAP), and endothelial factor 8. Sub confluent cell cultures of both control RPE and CNV RPE from the 5^th^ to the 12^th^ passage were used for patch-clamp experiments. Mean membrane capacitance of cultured cells was 128.9±17.8 pF (n=19); access resistance was 16.9±2.1 mega-Ω (n=19).

### Intracellular application of pp60^c-src^

During the whole cell recording, 30 U/ml pp60^c-src^ (>90% purity) was applied via the patch-pipette employing a procedure described by Wang and Salter [33]. In addition to pp60^c-src^ (Biomol, Hamburg, Germany), the pipette solution contained ATP (4x10^-3^ M) and the extracellular solution DIDS (5x10^-4^ M) to block Cl^-^ channels possibly activated by the application of ATP [34]. After the patch was opened into the whole-cell configuration, the maximal Ba^2+^ current amplitude was measured every 1 min and plotted over the experimental time normalized to the amplitude measured directly after breaking into the whole-cell configuration. As a control, a series of the same experiments were performed with heat-inactivated pp60^c-src^ (30 min at 95 °C).

### Immunoblotting

Membrane proteins were separated by polyacrylamide gel electrophoresis (8.5% polyacrylamide; Mini-Protean cells Bio-Rad Life Science Group, Hercules, CA; for 1 h at 150 V), and 30 mg of total protein were loaded in each lane. The proteins were blotted to nitrocellulose filter screens (Polyscreen, NEN®, Life Science Products Boston, MA) for 1 h at 100 V. Blots were blocked in phosphate buffered saline (PBS/Tween) with nonfat dry milk (10%) for 2 h and with bovine serum albumin (BSA; 5%) for 4 h at room temperature. They were then probed overnight at 4 °C with antibodies against Ca_v_1.2 and Ca_v_1.3 channel subunits and with the peroxidase-conjugated secondary antibody for 1 h at room temperature. Next, blots were visualized using a chemiluminescence kit (Amersham Pharmacia Biotech, Braunschweig, Germany) and digitized using the LAS-1000 Image Analyzer (Fujifilm, Berlin, Germany) and the AIDA 2.0 software (Raytest, Berlin, Germany). Specific staining was verified by stripping blots and staining them a second time using the same antibody together with the corresponding blocking peptide (antibody: blocking peptide, 1:1).

### Vascular endothelial growth factor secretion by retinal pigment epithelium cells

Confluent cell cultures were used to study VEGF secretion by RPE cells. For this assay 100,000 cells were kept in a chamber of a 12-well plate. Every chamber was equipped with 500 μl test solution (Dulbecco's modified Eagle medium with 0.5% fetal calf serum). Beginning with the last change of test medium, the concentration of VEGF-165 was measured every 4 h in the test medium using a commercially available ELISA kit (Biosource International, Solingen, Germany) according to the manufacturer's instructions. The number of experiments (n) includes the mean values of two repeated measurements of VEGF concentration in the medium in one experiment.

### Immunohistochemistry

Freshly isolated and cultured cells were stained with anti-class III β-tubulin antibody (Sigma). The following procedure was used for freshly isolated cells. After the perforated-patch recording of a pigmented cell the patch was opened into a classical whole-cell configuration, and negative voltage was applied to facilitate diffusion of the antibody from the pipette into the cell. The patch-pipette was removed from the cell, and the cell stayed attached to the ground and could be subjected to immuno-staining. In the first step, the cells underwent methanol-based fixation followed by permeabilization with Triton X-100 (0.5% in PBS). Then cells were washed several times and incubated with the secondary fluorescence conjugated antibody. Cultured cells underwent methanol-based fixation and permeabilization before they were incubated with the first antibody for 1 h. After being placed through a series of washes cells were next incubated with the secondary fluorescence conjugated antibody. Fluorescence staining was visualized by analysis (Soft Imaging System, Muenster, Germany).

Confluent cultures, which had been subjected to VEGF-A secretion assay, were tested for their homogeneity by means of immunohistochemistry methods as previously described in reference [31]. In brief, cultures were rinsed with PBS and fixed with acetone/PBS 1:1 followed with iced acetone. Cultures were incubated with antibodies against cytokeratin 18 (Sigma), vimentin, glial fibrillary acidic protein (GFAP), endothelial factor 8 (all from DAKO, Hamburg, Germany). Cell nuclei were counter-stained with hematoxylin. Primary antibodies were stained with alkaline phosphatase- or peroxidase-labeled secondary antibodies (Sigma) with fast red as substrate.

### Statistics

Numbers of experiments (n) with freshly isolated cells correspond to numbers of individual AMD patients or eyes without CNV. Data from cultured cells from CNV membranes were obtained from three different cultures from three different patients. All data are stated as mean±SEM. Data were tested for statistical significance by ANOVA (Sigma Plot, Systat Software, Inc., Point Richmond, CA). Significance was considered at p-values lower than 0.05.

## Results

### Voltage-dependent Ca^2+^ channels in freshly isolated cells

**Figure 2:**
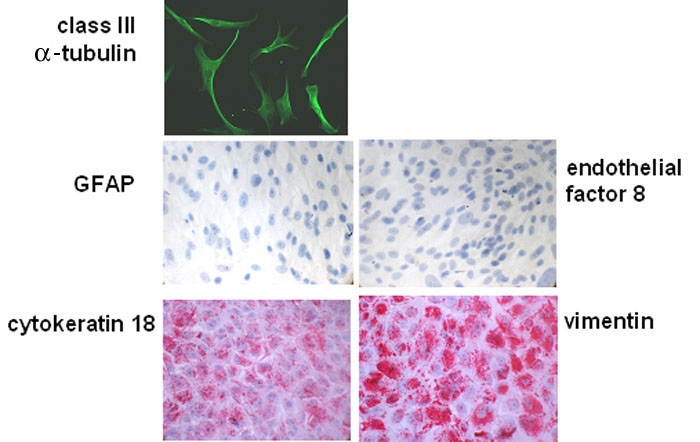
Analysis of retinal pigment epithelium cell cultures from CNV membranes. Sub-confluent cultures of retinal pigment epithelium (RPE) cells from choroidal neovascularization (CNV) tissues used for patch-clamp analysis were immunostained against class III β-tubulin. Confluent RPE cell cultures from CNV tissues were stained against glial acidic fibrillary protein (GFAP), endothelial factor 8, cytokeratin 18, or vimentin.

Cells were identified as RPE cells by subsequent immunohistochemical staining and fluorescence microscopy (Figure 1G, Figure 2). For this purpose, the solution of the recording pipette contained antibodies against class III β-tubulin, a marker for RPE cells in both healthy and several forms of disease [35]. Once the membrane currents were recorded, the antibody was intracellularly applied via dialysis by the solution in the patch-pipette.

**Figure 3:**
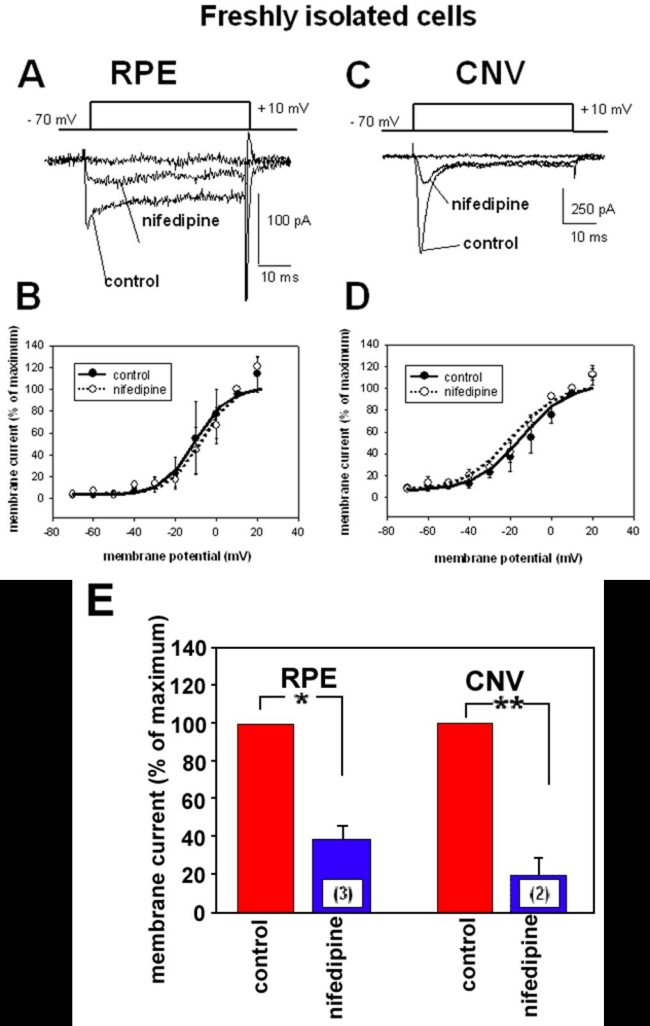
Effect of the dihydropyridine compound nifedipine on voltage-dependent Ba^2+^ currents of freshly isolated RPE cells. **A**: Ba^2+^ currents induced by a 50 ms voltage-step from -70 mV to +10 mV in a retinal pigment epithelium (RPE) cell freshly isolated from an eye without choroidal neovascularization (CNV) under control conditions and in the presence of nifedipine (10^-5^ M). **B**: Curves of voltage-dependent activation from six freshly isolated cells from eyes without CNV: three cells under control conditions and three cells in the presence of nifedipine. Currents were normalized to the maximal current amplitude and fitted using the Boltzmann equation. **C**: Ba^2+^ currents induced by a 50 ms voltage-step from -70 mV to +10 mV in an RPE cell freshly isolated from a CNV membrane under control conditions and in the presence of nifedipine (10^-5^ M). **D**: Curves of voltage-dependent activation from eight cells freshly isolated from CNV membranes: six cells under control conditions and two cells in the presence of nifedipine. Currents were normalized to the maximal current amplitude and fitted using the Boltzmann equation. **E**: Comparison of the inhibitory effect of nifedipine in freshly isolated RPE cells from eyes without CNV and from CNV tissues. Maximal currents at +10 mV under control conditions were set as 100%.

Freshly isolated RPE cells showed voltage-dependent inward currents in the absence of potassium. With Ba^2+^ as charge carrier, these cells responded to depolarization from a holding potential of -70 mV (Figure 1B) to values more positive than -30 mV with fast activating and inactivating inward currents (Figure 1B). The currents showed a maximal current density at +10 mV (Figure 1C) with 1.9±0.6 pApF^-1^ with a range from 1.4 to 2.8 pApF^-1^ (n=3; Figure 1F), a potential of the half maximal activation of -9.7±1.9 mV (n=3; Figure 3B), a time to peak of 9.7±2.8 ms (n=3), and an inactivation time constant of 53.9±10.3 ms (n=3).

In six out of 13 (46%) investigated CNV membranes, RPE cells showed voltage-dependent Ba^2+^ currents (Figure 1D-G). The mean current density of the maximal amplitude at +10 mV was 7.0±3.4 pApF^-1^ (n=6; current density ranged at +10 mV from 3.5 pApF^-1^ to 24.4 pApF^-1^), which was reached after 2.8±0.4 ms (n=6). This was faster than in cells from control eyes (p=0.024). The potential of the half maximal activation was -15.4±1.3 mV (n=6) which was significantly more negative than in cells from control eyes (p=0.0254). The currents inactivated with a time constant of 12.6±2.3 ms (n=6). Thus, inactivation was faster than in cells from control eyes (p=0.00094).

The activation threshold at about -30 mV indicates that the Ba^2+^ currents were in freshly isolated control cells and in cells from CNV tissue currents through high-voltage-activated (HVA) Ca^2+^ channels. Dihydropyridine nifedipine, a blocker of an HVA channel subtype, the L-type Ca^2+^channels, inhibited maximal current amplitudes to 39.4±6.3% (n=3) of control in freshly isolated RPE cells from eyes without CNV (Figure 3A,B,E). Application of nifedipine (10^-5^ M, Figure 3F,D,E) reduced maximal current amplitudes to 20.7±7.5% (n=2) of the control values in freshly isolated cells from CNV tissues. The voltage-dependence of Ba^2+^ currents was not changed in the presence of nifedipine in cells from CNV tissues as well as in cells from eyes without CNV (Figure 3B,D).

### Ba^2+^ currents in cultured cells

To further examine the cellular physiological characteristics of RPE cells, we developed cultures from CNV tissues by following the methods of Schlunck et al. [31]. In order to test these cultures on their origin and homogeneity, cultures were labeled with antibodies against vimentin, cytoskeratin 18, endothelial factor 8, and glial acidic fibrillary protein (GFAP). Cultures were homogeneously stained with antibodies against vimentin or cytokeratin 18 (Figure 2). No staining was observed using antibodies against GFAP or endothelial factor 8. Identity of cells used for patch-clamp recordings was confirmed by staining against class III β-tubulin (Figure 1G).

**Figure 4:**
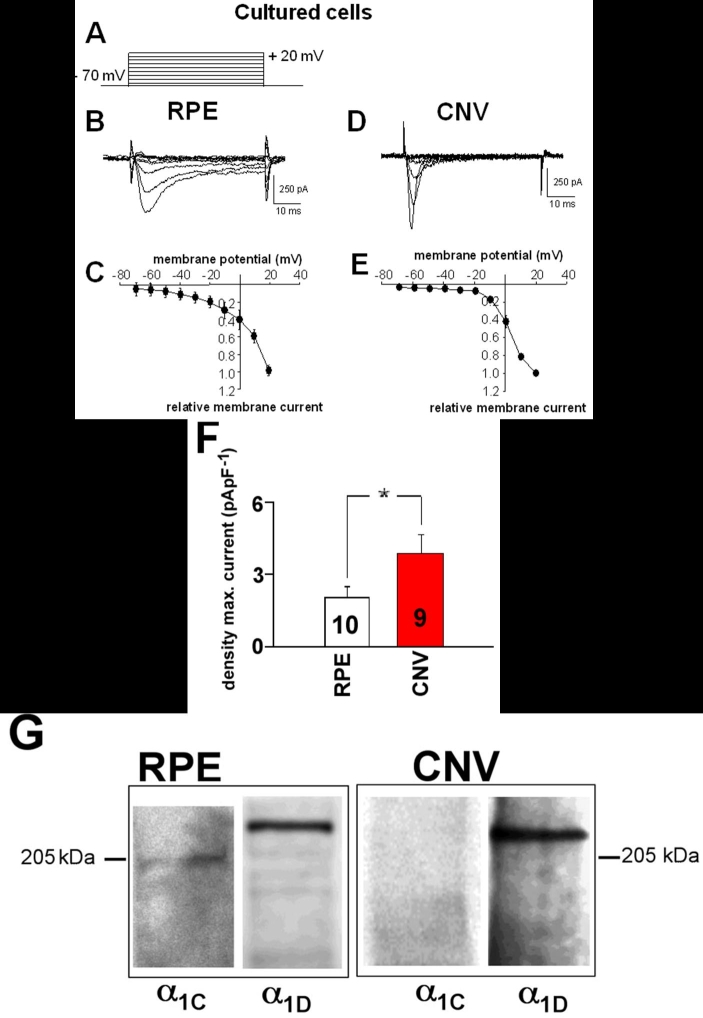
Voltage-dependent Ba^2+^ currents in cultured cells. **A**: Pattern of electrical stimulation to activate voltage-dependent Ba^2+^ currents. **B**: Ba^2+^ inward currents in a cultured human retinal pigment epithelium (RPE) cell of an eye without choroidal neovascularization (CNV). **C**: Current/voltage plot of currents from 10 cultured cells from eyes without CNV. **D**: Ba^2+^ inward currents in a cultured human RPE cell from CNV tissue. **E**: Current/voltage plot from nine cultured cells from CNV membranes. **F**: Comparison of maximal Ba^2+^ densities at +10 mV in cultured cells. **G**: Western blot of membrane proteins of cells from control eyes and CNV membranes. Left lane: staining for Ca_v_1.2 (channel α1C) subunits; right lane: staining for Ca_v_1.3 (channel α1D) subunits.

**Figure 5:**
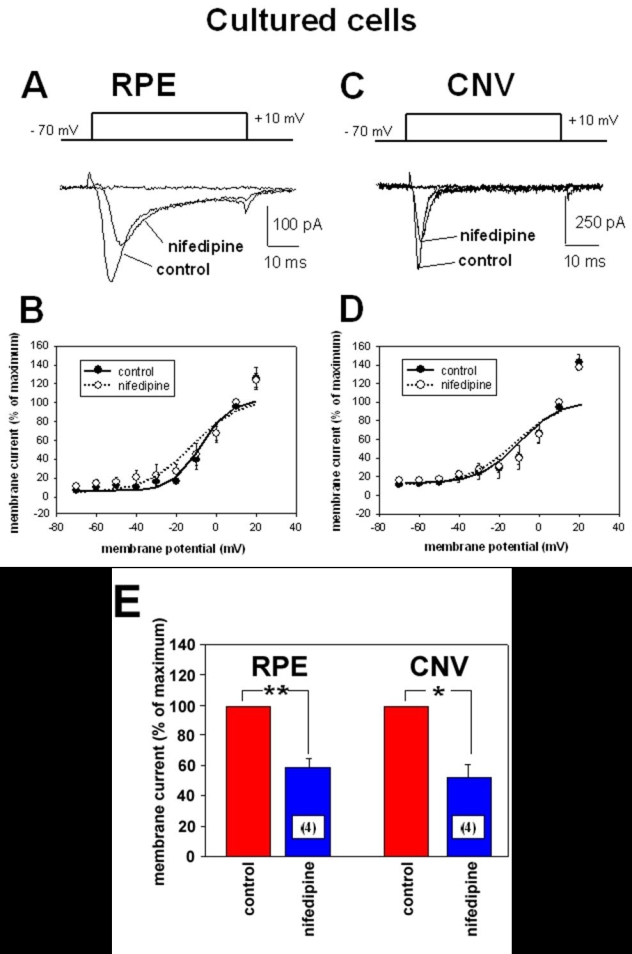
Effect of the dihydropyridine compound nifedipine on voltage-dependent Ba^2+^ currents of cultured retinal pigment epithelium cells. **A**: Ba^2+^ currents induced by a 50 ms voltage-step from -70 mV to +10 mV (upper panel) in a cultured retinal pigment epithelium (RPE) cell from an eye without choroidal neovascularization (CNV) under control conditions and in the presence of nifedipine (10^-5^ M). **B**: Curves of voltage-dependent activation from 14 cultured cells from eyes without CNV: 10 cells under control conditions and four cells in the presence of nifedipine. Currents were normalized to the maximal current amplitude and fitted using the Boltzmann equation. **C**: Ba^2+^ currents induced by a 50 ms voltage-step from -70 mV to +10 mV in a cultured RPE cell from a CNV membrane under control conditions and in the presence of nifedipine (10^-5^ M). **D**: Curves of voltage-dependent activation from 13 cultured cells from CNV membranes: nine cells under control conditions and four cells in the presence of nifedipine. Currents were normalized to the maximal current amplitude and fitted using the Boltzmann equation. **E**: Comparison of the inhibitory effect of nifedipine in cultured RPE cells from eyes without CNV and from CNV tissues. Maximal currents at +10 mV under control conditions were set as 100%.

RPE cells maintain the activity of voltage-dependent Ca^2+^ channels in culture (Figure 4 and Figure 5). The maximal current density at +10 mV in cultured RPE cells from CNV membranes was 3.9±0.8 pApF^-1^ (n=9) and in cultured RPE cells from eyes without CNV (2.0±0.4 pApF^-1^, n=10; p=0.049). Cells from CNV membranes had a time to peak of with 3.3±0.45 ms (n=9), which was faster than in cells from eyes without CNV (11.9±1.4 ms, n=10; p=0.00004). Western blot analysis of membrane proteins revealed the expression of Ca_v_1.3 subunits in cultured RPE cells from eyes without CNV and in cells from CNV membranes. A weak expression of Ca_v_1.2 subunits could only be detected in RPE cells from eyes without CNV (Figure 4G).

The currents in cultured cells were sensitive to the dihydropyridine nifedipine, too (Figure 5). Application of nifedipine reduced the maximal current amplitude measured at +10 mV to 53.1±8.7% (n=4) in cells from CNV tissues and to 60.1±4.8% (n=4) in cells from eyes without CNV. Furthermore, as in freshly isolated cells, the voltage-dependence of Ba^2+^ currents was not influenced by nifedipine in both cells from CNV tissues and cells from control eyes.

### Vascular endothelial growth factor secretion

**Figure 6:**
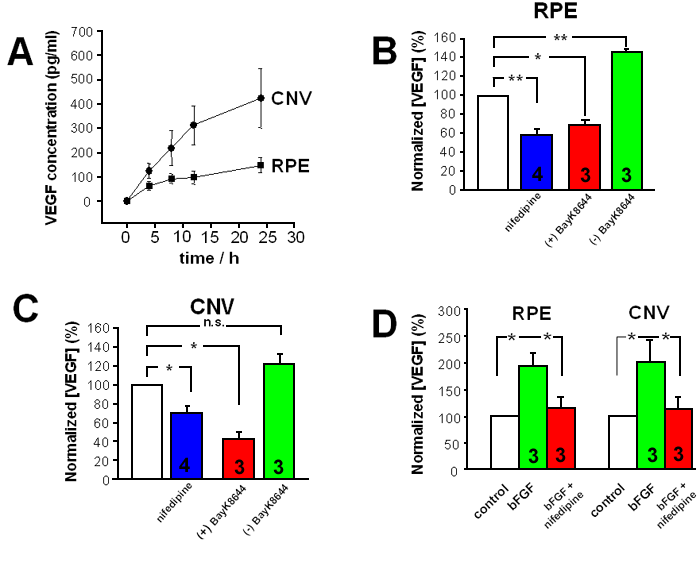
Vascular endothelial growth factor secretion. **A**: Increase of vascular endothelial growth factor (VEGF) concentration in the test solution over time (each group: n=3 which represent 3 cultures from 3 different patients). **B**: Effect of L-type Ca^2+^ channel modulation (inhibition: nifedipine, (+)BayK8644 enantiomer; activation: (-)BayK8644 enantiomer) on VEGF secretion by cells from eyes without CNV (8 h values). **C**: Effect of L-type channel modulation (inhibition: nifedipine, 10^-5^ (+)BayK8644 enantiomer; activation: 10^-5^ (-)BayK 8644 enantiomer) on VEGF secretion rate in cells from choroidal neovascularization (CNV) membranes (8 h values). **D**: Effect of nifedipine on basic fibroblast growth factor (bFGF) induced rise in the VEGF secretion rate by cells from eyes without CNV (left bars) and from CNV membranes (right bars; 8 h values).

**Figure 7:**
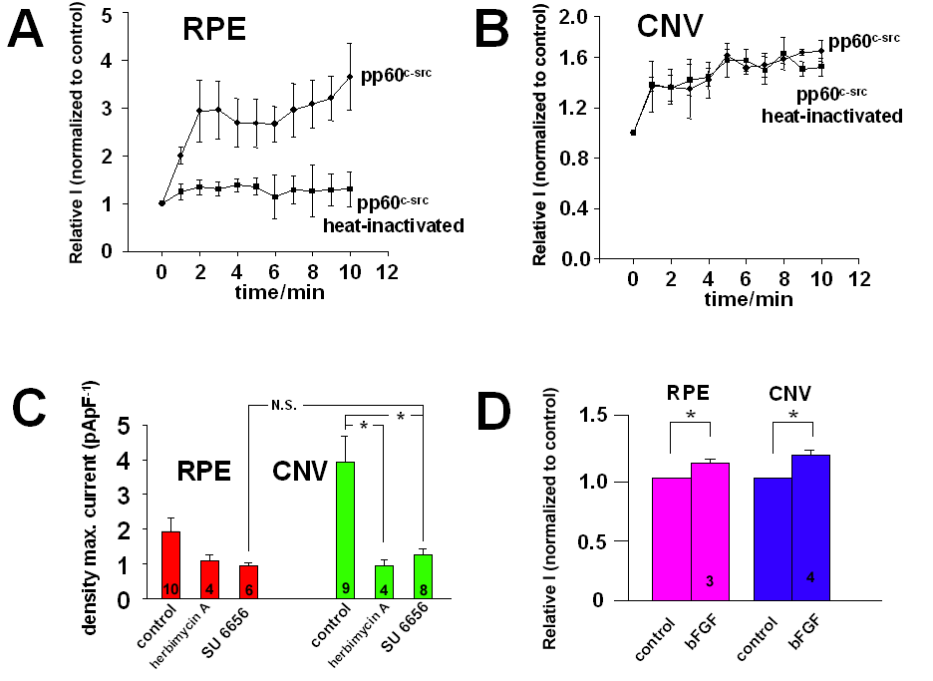
Regulation of L-type channels. **A**: Intracellular application of pp60^c-src^ (30 U/ml) via the patch pipette in cultured cells from eyes without choroidal neovascularization (CNV): change in maximal currents amplitudes by active pp60^c-src^ versus heat-inactivated pp60^c-src^. **B**: Intracellular application of pp60^c-src^ via the patch pipette in cultured cells from CNV membranes: change in the maximal current amplitudes by active pp60^c-src^ versus heat-inactivated pp60^c-src^. **C**: Effect of the src-kinase inhibitors herbimycin A (10^-5^ M) and SU6656 (10^-6^ M) on current density in cultured cells from eyes without CNV and from CNV membranes. **D**: Effect of basic fibroblast growth factor (bFGF) application (10 ng/ml) on L-type currents in cultured cells from eye without CNV and from CNV membranes. Control represents current amplitude before bFGF application, bFGF represents current amplitude after bFGF application.

Cultured RPE cells release VEGF into the medium, a process that could be stimulated by a test medium containing 0.5% fetal calf serum. Under these conditions, we found that VEGF concentration increased with time in the test medium in both RPE cells from eyes without CNV and in RPE cells from CNV membranes. However, the VEGF concentration increased significantly faster and reached higher concentrations in cultures containing cells from CNV membranes when compared to cultures of cells from eyes without CNV (Figure 6A). This resulted in a higher VEGF secretion rate of 12.8 ± 1.4 pg/h (versus no CNV: 5.4±0.9 pg/h; n=4; p=0.0475). Inhibition of Ca^2+^ channels by nifedipine (10^-5^ M) reduced VEGF secretion by both cell populations (Figure 6B,C). To confirm the conclusion that activation of voltage-dependent Ca^2+^ channels leads to secretion of VEGF, we used the L-type channel Ca^2+^ channel modulator BayK8644 (10^-5^ M; Figure 6B,C) as (-) enantiomer, which activates L-type channels and as the (+) enantiomer, which inhibits L-type channels. The (-) BayK8644 increased the VEGF secretion rate by RPE cells from eyes without CNV (Figure 6B), whereas it only slightly increased the VEGF-A secretion rate in cells from CNV membranes (Figure 6C). The latter effect was not statistically significant. The (+) BayK8644 reduced the VEGF-A secretion rate in both RPE cells from CNV membranes (Figure 6C) as well as in cells from eyes without CNV (Figure 6B). To achieve a more realistic stimulation of RPE cells, we used a test medium that contained bFGF (10 ng/ml). In both, cells from CNV tissue and cells from eyes without CNV, bFGF increased the VEGF secretion rate. Further inhibition of voltage-dependent Ca^2+^ channels by nifedipine reduced the bFGF-induced increase in the VEGF secretion rate (Figure 6D) to control values. In addition, bFGF increased L-type channel activity in RPE cells from CNV membranes as well as in cells from eyes without CNV (Figure 7D).

### Regulation of voltage-dependent Ca^2+^ channels

Since the voltage-dependent Ca^2+^ channels mediate growth factor-dependent secretion of VEGF, we studied the regulation of these ion channels to find a possible link to how growth factors stimulate VEGF secretion by stimulation of Ca^2+^ channels. Intracellular application of the cytosolic subtype of tyrosine kinase pp60^c-src^ (30 U/ml) during the whole-cell configuration via the patch-pipette increased the maximal current amplitude of L-type channels in RPE (n=4; Figure 7A). Application of heat-inactivated pp60^c-src^ did not change the L-type channel activity (n=4). Furthermore, intracellular application of pp60^c-src^ accelerated time-dependent inactivation of L-type channel currents (reduction of inactivation time constant to 22±11% of control measured directly after breaking into the whole-cell configuration; n=7; p=0.00036 paired test) Thus, pp60^c-src^ subtype of tyrosine kinase is an activator of L-type channel activity and modulator of L-type channel kinetics in human RPE cells. Intracellular application of the active pp60^c-src^ to RPE cells from CNV membranes did not increase the maximal current amplitude (n=3; Figure 7B) or change the time-dependent inactivation (inactivation time constant 67±21% of control; n=4; not significant with paired test). However, inhibition of pp60^c-src^ by overnight incubation with herbimycin A (10^-5^ M) or by 1 h incubation with SU6656 (10^-6^ M, according to [36,37]) reduced the current density to the same base level in both groups of cells. (Figure 7C). Thus, L-type channels in cells from CNV membranes are activated by src-subtype tyrosine kinase, but these channels cannot be further stimulated by pp60^c-src^. However, as we demonstrated bFGF can further stimulate L-type channel activity in cells from CNV membranes (Figure 7D).

## Discussion

The RPE is known to secrete a variety of growth factors [5-7,38]. In this study, we provide insight into the mechanisms that regulate their secretion rate. We found the rate of VEGF secretion by human RPE cells is dependent on the activation of L-type Ca^2+^ channels. Furthermore, RPE cells from CNV tissue also maintained expression of voltage-dependent Ca^2+^ channels and secreted the angiogenic factor VEGF in response to activation of these channels. Our observations might be of importance to explore new strategies to prevent or reduce CNV.

Freshly isolated human RPE cells showed dihydropyridine-sensitive voltage-dependent Ba^2+^ inward currents in response to depolarization to potentials more positive than -30 mV from a holding potential of -70 mV. Thus, fresh human RPE cells exhibited currents with properties of L-type Ca^2+^ channels. We used a mixture of charge carrier ions of 1 mM Ca^2+^ and 10 mM Ba^2+^ to enhance seal formation and stability of the perforated-patch configuration. This would lead to an anomalous mole fraction effect [39-41]. This effect arises from the higher affinity of Ca^2+^ compared to Ba^2+^ at the selectivity filter of the Ca^2+^ channel pore and leads to changes in the current properties such as smaller amplitudes or even occasional shifts in the voltage-dependence of the currents [39,41]. However, the presence of L-type channels is in accordance with many studies using freshly isolated or cultured RPE cells from various species [10-15,18,42,43]. The L-type channel currents in freshly isolated human RPE cells exhibited fast time-dependent activation, rather negative potential of half-maximal activation, and were reduced to only 40% of the control values in the presence of 10^-5^ M nifedipine, a rather large concentration. With the low dihydropyridine sensitivity, voltage-dependence, and kinetic behavior, the currents in freshly isolated human RPE cells share characteristics with currents exhibited by L-type Ca^2+^ channel α-subunit Ca_v_1.3 investigated in a heterologous expression system [19-21]. The dihydropyridine block was incomplete in which the currents differ from those in heterologous expression system. However, characterization of Ca_v_1.3 channels in native cells revealed that these channels were also only incompletely blocked by dihydropyridines at large concentrations [44]. Thus, the currents in freshly isolated RPE cells from eyes without CNV display characteristics of Ca_v_1.3 channels by means of voltage-dependence, dihydropyridine sensitivity, and activation kinetics. The application of nifedipine did not change the voltage-dependent activation of the channels. Thus, the L-type channel blocker did not unmask another Ca^2+^ channel with a different voltage-dependence contributing to the whole-cell Ba^2+^ currents. The expression of the Ca_v_1.3 subunit was confirmed by Western blot analysis of membrane proteins. The predominant expression of Ca_v_1.3 subunits in the RPE was also described in rat and mouse cells [12,15,42,43]. However, the L-type channel currents in human RPE cells showed a fast inactivation with a time constant of 50 ms. Ca_v_1.3 subunits in a heterologous expression system showed almost no inactivation [19,20] except in one study [21]. The reason for this difference remains to be elucidated. It might be due to the absence of an anomalous mole fraction effect in other studies that did not use extracellular solutions containing both Ba^2+^ and Ca^2+^, to different phosphorylation in native cells, or different composition of accessory subunits. Another possibility might be the specific splice variant expressed in RPE cells [42]. This splice variant has not been so far described to be present in other tissues, and its characteristics are unknown.

The western blot analysis analysis also revealed a weak expression of Ca_v_1.2 (channel α1C) subunits. The time- and voltage-dependent activation indicates that this subtype of L-type channel might only weakly contribute to the whole-cell Ba^2+^ currents in RPE cells, but it cannot be excluded.

The expression of Ca_v_1.3 (or α1D subunits) known as neuroendocrine L-type channels [16] in RPE cells implies a role in the control of the secretory activity. Since expression of L-type Ca^2+^ channels was maintained in cultured cells, we analyzed effects of L-type channel modulation on VEGF secretion by RPE cells in vitro. Direct modulation of L-type channels by application of the dihydropyridine compound BayK8644 substantially changed the VEGF secretion rate. Application of the Ca^2+^ channel activating enantiomer of BayK8644 increased the VEGF secretion rate. The same was observed by stimulation with low concentration of fetal calf serum. Furthermore, application of bFGF stimulated both L-type channel currents and VEGF secretion rate. The growth-factor-stimulated increase in VEGF secretion was reduced by L-type channel inhibition using nifedipine or the Ca^2+^ channel blocking enantiomer of BayK8644. Thus, either direct or growth-factor-dependent stimulation of L-type channels resulted in an increase of the VEGF secretion rate. The underlying growth-factor-dependent increase in L-type channel activity might be achieved in two ways. First, the bFGF receptor FGFR-2 can stimulate L-type channels by direct interaction with the Ca_v_1.3 subunit [15]. Second, the Ca_v_1.3 subunit in rat RPE was found to directly interact with the cytosolic tyrosine kinase pp60^c-src^ [12]. In human cells pp60^c-src^ inhibition results in a decrease of L-type channel activity, whereas intracellular application of pp60^c-src^ leads to an increase of L-type channel activity. Thus, pp60^c-src^ constitutively maintains a basic level of L-type channel activity, which can be further increased. Since stimulation by several growth factors results in an increase of pp60^c-src^ activity, the growth-factor-dependent stimulation of VEGF secretion might result from pp60^c-src^-dependent activation of L-type channels.

Patch-clamp recordings with freshly isolated cells from CNV revealed that RPE cells can maintain the expression of voltage-dependent Ca^2+^ channels in disease. In a number of investigated membranes, none of the RPE cells displayed any voltage-dependent current. These cells showed a linear current/voltage relationship with maximal current amplitudes in the range of +/- 20 pA. This corresponds with observations in histological investigations of CNV membranes. In these studies, RPE cells in CNV membranes appeared as atrophic cells, a consequence of a disease that starts in the RPE [9]. However, we found that other CNV membranes contained RPE cells appearing to be functional active cells. These cells expressed voltage-dependent Ca^2+^ channels. The currents activate at relatively positive potentials, showed fast activation kinetics and were inhibited by the dihydropyridine nifedipine. With these characteristics, the currents were comparable with those in cells from eyes without CNV, mostly L-type channel currents. However, currents in cells from CNV membranes showed unusual fast inactivation kinetics, and the potential of half-maximal activation was shifted to more negative potentials. Thus, it could be that in these cells another voltage-dependent Ca^2+^ channel contributes to the observed Ba^2+^ currents. Since application of nifedipine resulted in significant current inhibition without shifts in the voltage-dependent activation, the presence of nifedipine did not unmask the contribution by another Ca^2+^ channel with different voltage-dependence to the whole-cell currents. Furthermore, we could also detect the presence of Ca_v_1.3 subunits in RPE cells from CNV tissues. Thus, it is likely that these L-type channels contribute, at least in part to the Ba^2+^ currents in these cells. In all cells from CNV membranes, the voltage-dependent activation was shifted to more negative values, and the currents reached their maximum more rapidly. Thus, in RPE cells from CNV membranes, smaller depolarizing shifts in membrane potential led to faster activation of L-type channels, which, in turn, generated larger Ca^2+^ influxes into the cell. These differences could be again due to an anomalous mole fraction effect on different Ca^2+^ channel subtypes [39,41]. Another reason for this might be a higher pp60^c-src^-dependent stimulation of L-type channels. CNV tissue represents a growth-factor-rich environment [5,6,8,27,45] that should increase the pp60^c-src^ activity in general. Furthermore, intracellular application of pp60^c-src^ to RPE cells from control eyes resulted in faster inactivation of L-type channel currents and has been found to shift the potential of the half-maximal activation to more negative values in rat RPE cells [12]. Since intracellular application of pp60^c-src^ did not further increase L-type channel activity in RPE cells from CNV membranes, it appears that L-type channels in these cells are maximally stimulated by this tyrosine kinase. This is supported by the observation that inhibition of pp60^c-src^ in cells from CNV membranes reduced the L-type channel activity to the same level as in cells from control eyes.

RPE cells from CNV membranes not only maintained the expression of nifedipine-sensitive voltage-dependent Ca^2+^ channels, but the VEGF secretion rate is also dependent on the activity of these channels. Application of nifedipine or the Ca^2+^ channel inhibiting (+) enantiomer of BayK8644 significantly reduced the VEGF-A secretion to the same level. The Ca^2+^ channel activating (-) enantiomer of BaK8644 slightly, but not significantly, increased VEGF-A secretion by RPE cells from CNV membranes. This is not unlike experiments where intracellular application of src-kinase was used to stimulate Ca^2+^ channel activities in CNV RPE cells. Here, also, no further stimulatory effect was observed. Several angiogenic and growth factors are present in CNV membranes. Vitronectin [46], a major component of drusen [47,48], VEGF [49] via KDR/flk-1 receptor expressed by RPE cells [50,51], or IGF-1 [25-27,52,53] act via activation of the src-subtype tyrosine kinase, and many are produced by the RPE [5-7,22-27] and bFGF [54]. We propose that the secretion of these factors is regulated by L-type channels at the beginning of the disease. In the progression of the disease growth of CNV tissue is promoted by VEGF-A secretion under control of nifedipine-sensitive voltage-dependent Ca^2+^ channels. The CNV membranes, which were found to contain RPE cells with active Ca^2+^ channels, might reflect a state of active CNV development. Ca^2+^ channel activity and VEGF secretion rate appears to be higher in cells from CNV tissues compared to cells from eyes without CNV. Immunohistological analysis of RPE cell cultures from CNV tissues revealed that these cultures contained homogeneous RPE cells. RT-PCR analysis revealed the expression of the RPE marker RPE65 in these cultures [31]. The presence of endothelial cells could be excluded by absence of endothelial factor 8 staining, and the presence of glia cells could by excluded by absence of GFAP staining. It is unlikely that cultured photoreceptors or neurons were present.

It is intriguing to argue that this might be a pathophysiological effect. However, this could also be an effect resulting from a different reaction to the experimental procedures. In contrast, taking the different Ca^2+^ channel properties into account it might be that RPE cells in the proliferating CNV tissue underwent a transdifferentiation into a different phenotype, which is characterized by predominant growth factor secretion. This phenotype is maintained in culture as can be seen by the conserved Ca^2+^ channel characteristics. As already stated, these characteristics could be due to the expression of additional Ca^2+^ channel subtypes.

In summary, we showed for the first time that regulation of the angiogenic factor secretion rate by human RPE cells involves the activation of voltage-dependent L-type Ca^2+^ channels. Since a changed growth factor secretion by the RPE is thought to play a role in the induction of various eye diseases involving cell proliferation, voltage-dependent Ca^2+^ channels or their regulatory proteins could be included as targets in therapeutic strategies acting directly at the secretion site of angiogenic factors.
